# Que Va a la Chasse Perd sa Place

**Published:** 1863-05

**Authors:** 


"Que va à la chasse perd sa place."—This proverb has had a somewhat amusing literal fulfilment lately at Dolmabaktche. On Sunday the Sultan was afflicted by an attack of severe toothache, and a messenger was accordingly despatched to summon M. Roux, his Majesty's dentist, for the treatment of the imperial molar. The fashionable tooth-doctor was not to be found; he had gone *a la chasse;* and though mounted messengers beat him up for nearly three hours round all the "covers," from Baluky to far beyond Meslak, he was nowhere to be found. Chamberlains of high and low degree were at their wit's ends, when the happy recollection struck somebody that there was another knight of the forceps—unknown, indeed, to fame, but still professing the art and mystery of tooth-drawing, in a garret opposite Galata-Serai. *A ferim!* off went a mounted messenger for the man of science, and without time given him to make his neglected ablutions, or borrow an unragged surtout, the bewildered operator was whisked away to the palace. Urgent however, as was the need of his services, it was found necessary to subject him to a process of toilette before he could be ushered into the suffering presence. This was done as rapidly as half-a-dozen valets could perform it, and in a few minutes the offending grinder was extracted—fortunately without damage to the imperial jaw. The operation over, his Majesty questioned Mr. Z. as to his personal belongings, and finding that bad luck and short commons had been his lot for years past, resolved at once to force fortune into better humor on his behalf. Without hypercritical curiosity as to diplomas or other professional vouchers, he at once named him special dentist to himself, with a salary of 1600p. a month, an immediate *cadeau* of 150 liras, and an excellent house at Ortakeni.

*Moral.*—Dentists (and doctors) should always be found at the post of duty.

